# The Role of *Pseudomonas aeruginosa* ExoY in an Acute Mouse Lung Infection Model

**DOI:** 10.3390/toxins10050185

**Published:** 2018-05-04

**Authors:** Christina Kloth, Bastian Schirmer, Antje Munder, Tane Stelzer, Justin Rothschuh, Roland Seifert

**Affiliations:** 1 Institute of Pharmacology, Hannover Medical School, 30625 Hannover, Germany; kloth.christina@mh-hannover.de (C.K.); tst@posteo.de (T.S.); rothschuh.justin@mh-hannover.de (J.R.); seifert.roland@mh-hannover.de (R.S.); 2 Clinic for Paediatric Pneumology, Allergology and Neonatology, Hannover Medical School, 30625 Hannover, Germany; munder.antje@mh-hannover.de; 3 Institute of Experimental Hematology, Hannover Medical School, 30625 Hannover, Germany; 4 Biomedical Research in Endstage and Obstructive Lung Disease Hannover (BREATH), German Center for Lung Research, 30625 Hannover, Germany

**Keywords:** *Pseudomonas aeruginosa*, bacterial effector protein, nucleotidyl cyclase, lung infection, T3SS

## Abstract

The effector protein Exotoxin Y (ExoY) produced by *Pseudomonas aeruginosa* is injected via the type III secretion system (T3SS) into host cells. ExoY acts as nucleotidyl cyclase promoting the intracellular accumulation of cyclic nucleotides. To what extent nucleotidyl cyclase activity contributes to the pathogenicity of ExoY and which mechanisms participate in the manifestation of lung infection is still unclear. Here, we used an acute airway infection model in mice to address the role of ExoY in lung infection. In infected lungs, a dose-dependent phenotype of infection with bacteria-expressing ExoY was mirrored by haemorrhage, formation of interstitial oedema in alveolar septa, and infiltration of the perivascular space with erythrocytes and neutrophilic granulocytes. Analyses of the infection process on the cellular and organismal level comparing infections with *Pseudomonas aeruginosa* mutants expressing either nucleotidyl cyclase-active or -inactive ExoY revealed differential cytokine secretion, increased prevalence of apoptosis, and a break of lung barrier integrity in mice infected with cyclase-active ExoY. Notably, of all measured cyclic nucleotides, only the increase of cyclic UMP in infected mouse lungs coincides temporally with the observed early pathologic changes. In summary, our results suggest that the nucleotidyl cyclase activity of ExoY can contribute to *P. aeruginosa* acute pathogenicity.

## 1. Introduction

The Gram-negative bacterium *Pseudomonas aeruginosa* (*P. aeruginosa*) is an important opportunistic pathogen that preferentially infects patients suffering from a compromisedh immune status or a chronic lung disease [1,2,3,4,5]. As *P. aeruginosa* has a broad variety of virulence factors and is able to establish biofilm-protected niches, elimination of the bacterium is a central medical concern not only restricted to chronically ill patients [6,7].

One of the bacterial virulence factors is the type III secretion system (T3SS), which enables the bacterium to inject the associated effector proteins ExoS, ExoT, ExoU, and ExoY into eukaryotic host cells via a needle-like structure [8]. These effector proteins affect different intracellular signal transduction pathways resulting in cellular dysfunction and, ultimately, in cellular death [9]. Both ExoS and ExoT are GTPase-activating proteins and ADP ribosyltransferases affecting epithelial integrity and viability. ExoU exhibits a phospholipase A_2_ activity leading to cell necrosis. However, the role of ExoY during *P. aeruginosa* infections is poorly understood. ExoY has been originally described as soluble adenylyl cyclase depending on at least one cytosolic proteinaceous cofactor for full enzymatic activity [10]. Recently, F-actin has been shown to bind ExoY and to stimulate adenylyl and guanylyl cyclase activity of the toxin [11]. The described effects of ExoY suggest a distinct pathogenic function of ExoY in *P. aeruginosa* infections. However, some published data show no significant in vivo cytotoxic effect of ExoY [12,13,14]. Several studies with genetically-engineered *Pseudomonas* mutants lacking genomically-encoded T3SS effectors, but expressing a plasmid-encoded ExoY [10], have been published [15,16,17]. In these studies, a distinct phenotype after infection with the mutants could be demonstrated. A closer look at the literature reveals that all studies which have shown distinct in vivo effects of ExoY have been performed using these genetically-constructed *Pseudomonas* mutants. Stevens et al. observed a more severe lung injury and significantly increased endothelial permeability in rat lungs infected with catalytically-active ExoY as compared to an inactive ExoY mutant [17]. To date, none of these effects have been demonstrated with naturally-occurring ExoY-expressing strains [12,13,14]. However, it seems unlikely that there is no function of ExoY in *P. aeruginosa* infections, since the protein is expressed in 89% of clinical *P. aeruginosa* isolates and environmental strains—as compared to the lower abundance of the “harsh” exotoxins ExoS (70%) and ExoU (28%) [18]. 

We hypothesize that ExoY modulates the course of acute lung infection and that the toxin exerts its pathogenetic effects by targeting the integrity of the lung epithelium and facilitating the penetration of bacteria into the parenchyma. Thus, in this study we aim at investigating the precise time- and dose-dependent phenotype of lung infection. Moreover, we intended to confirm key findings observed in rats [17] in our well-established mouse lung infection model [14,19]. For this purpose, we used a genetically-engineered *P. aeruginosa* strain harbouring either ExoY (ExoY) or a catalytically-inactive ExoY mutant (ExoY^K81M^) as the only T3SS effector protein. With this study, we provide the first phenotypic and basic immunological description of ExoY effects in a standardised mouse model. Furthermore, we directly compared different infection doses and focussed on the early infection (0–48 h), thus complementing previous investigations of later time points. 

## 2. Results

### 2.1. Distinct In Vivo Phenotypes after Infection with the ExoY Mutants

**Figure 1:**
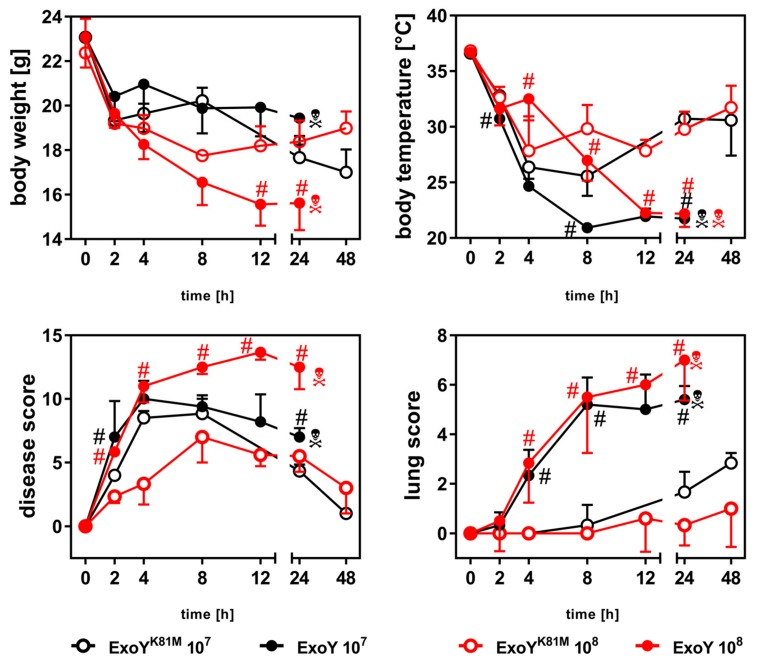
Phenotype of infected mice. Time- and dose-dependent development of body temperatures, body weights, disease scores and lung scores of infected mice. Shown are the means ± SD of *n* = 6 animals. Differences between ExoY and ExoY^K81M^ of the same infection dose (#) were considered significant when *p* ≤ 0.05 (multiple *t*-test with post-hoc Holm-Sidak correction).

**Figure 2:**
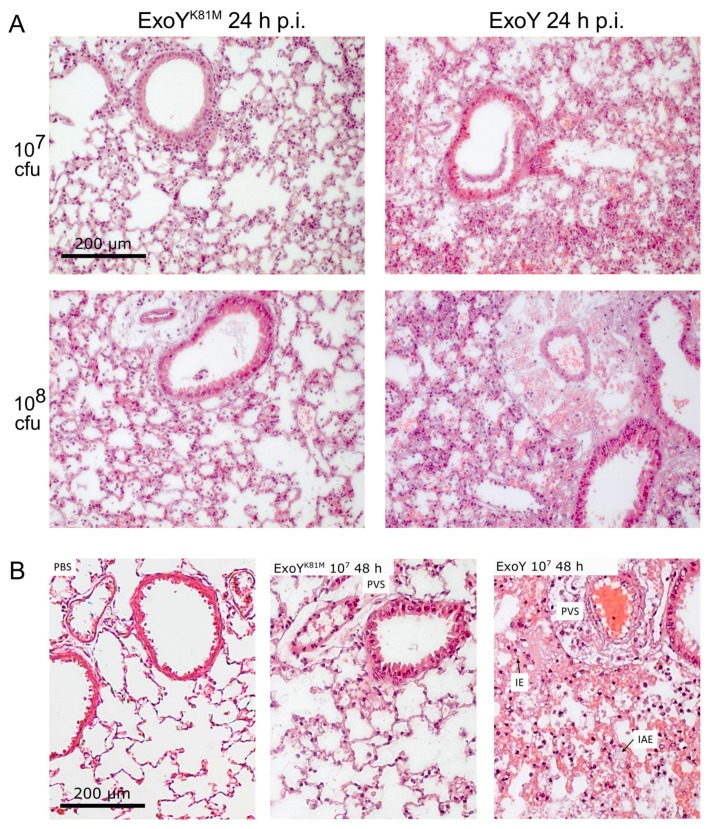
Histologic alterations in infected lungs. (**A**) Representative micrographs of ExoY-/ExoY^K81M^-infected lung tissue at 24 h after infection (For a corresponding micrograph of mock-infected mice lungs, see Supplementary Figure S4). All infected mice showed intra-alveolar haemorrhage, interstitial oedema in alveolar septa, and infiltration of the perivascular space with erythrocytes and granulocytes, but in ExoY^K81M^-infected mice, a marked reduction of these phenomena as compared to the dose-matched ExoY group was observed. The extent of the observed alterations was comparable in both infection doses. Standard H and E staining, scale bar = 200 µm. (**B**) Micrographs of lung tissue from mock-infected (PBS), ExoY^K81M^- or ExoY-infected mice 48 h after infection. Standard H&E staining, scale bar = 200 µm. Normal appearance of alveolar septa and perivascular space (PVS) in mock-infected lung tissue or after infection with ExoY^K81M^ bacteria. Interstitial oedema (IE) in alveolar septa, intra-alveolar oedema (IAE), alveolar haemorrhage and infiltration of perivascular space (PVS) with blood and granulocytes occur after infection with ExoY bacteria.

**Figure 3:**
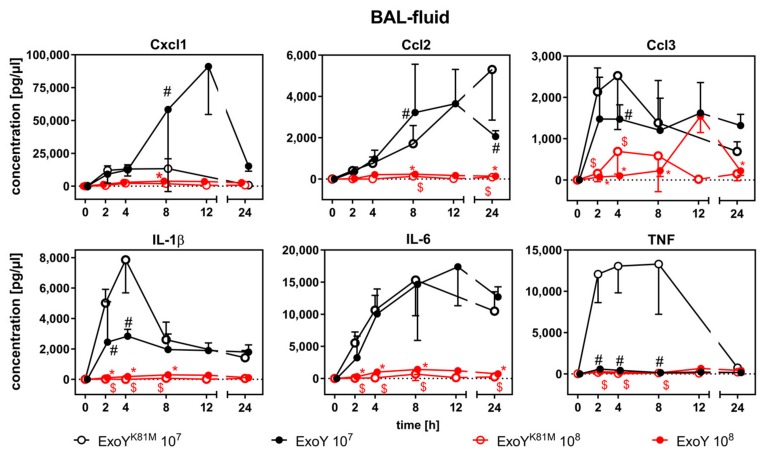
Expression of inflammatory cytokines in BAL-f of mice after infection with ExoY^K81M^ and ExoY. Data represent the mean ± SD of *n* = 6 animals. Overall cytokine concentrations were lower in mice infected with 10^8^ cfu, where no difference between Exo and ExoY^K81M^ was observed. In mice infected with 10^7^ cfu, the local immune response was comparable in terms of IL-6 concentration, but dampened in terms of TNF, IL-1β, and CCL3 concentrations. Differences between ExoY infection doses (*), between ExoY^K81M^ infection doses ($), and between ExoY and ExoY^K81M^ of the same infection dose (#) were considered significant when *p* ≤ 0.05 (two-way-ANOVA with post-hoc Holm-Sidak correction).

**Figure 4:**
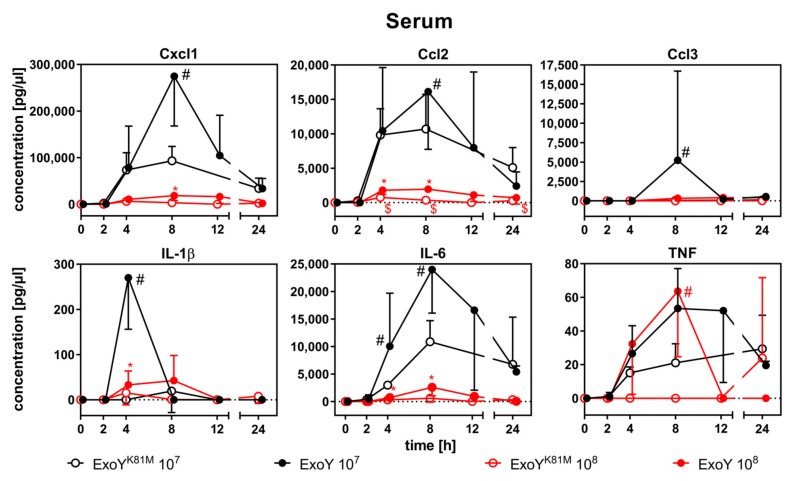
Expression of inflammatory cytokines in sera of mice after infection with ExoY^K81M^ and ExoY. Overall cytokine concentrations were lower in mice infected with 10^8^ cfu, where only TNF was significantly increased at 8 h in ExoY-infected mice as compared to ExoY^K81M^. In mice infected with 10^7^ cfu, the systemic immune response was increase in terms of Cxcl2, Ccl2, Ccl3, IL-1β, IL-6, and TNF concentrations in sera. Data represent the mean ± SD of *n* = 6 animals. Differences between ExoY infection doses (*), between ExoY^K81M^ infection doses ($), and between ExoY and ExoY^K81M^ of the same infection dose (#) were considered significant, when *p* ≤ 0.05 (two-way-ANOVA with post-hoc Holm-Sidak correction).

We compared the time course of infection in mice inoculated with ExoY to that in mice infected with the cyclase-inactive mutant ExoY^K81M^. Two different infection doses (10^7^ and 10^8^ cfu/mouse) were used. Parameters to be analysed were body temperatures and weights, as well as disease and lung scores (Figure 1), histological alterations (Figure 2A,B) and cytokine concentrations in bronchoalveolar lavage fluids (BAL-f) (Figure 3) and sera (Figure 4). 

Body weights of all infected mice decreased between 0–8 h. Mice infected with 10^8^ cfu ExoY^K81M^ showed a beginning recovery of body weight starting from 8 h p.i., whereas body weights of the other experimental groups declined further (10^8^ cfu ExoY, 10^7^ cfu ExoY^K81M^) or stagnated (10^7^ cfu ExoY). Body temperature of all infected mice decreased and reached a minimum between 4–8 h. Whereas mice infected with ExoY^K81M^ started to regain normal body temperatures at 8–12 h, mice infected with ExoY did not. Strikingly, mice infected with 10^8^ cfu ExoY exhibited a temporary stagnation of body temperature decrease between 2 and 4 h. All of the ExoY-infected, but none of the ExoY^K81M^-infected, mice had to be sacrificed due to severe clinical symptoms within 48 h. The disease scores of all infected mice increased drastically during the first hours of infection with a maximum score at 4 h (ExoY, 10^7^) or 8 h (ExoY^K81M^, 10^7^ and 10^8^). Mice infected with 10^8^ cfu ExoY showed a continuous progression of disease scores, whereas all other groups displayed a progredient amelioration of disease signs after an intermittent peak. While all experimental groups exhibited significant signs of infection regarding the overall disease score, only mice infected with ExoY showed a significant increase in lung pathology scores as soon as 2 h after infection. With a latency of around 6 h mild signs of lung pathology started to evolve in the ExoY^K81M^ groups, too (Figure 1). 

Pathophysiological changes in the lungs after infection with the ExoY bacteria were particularly detectable by microscopic investigation of the tissue. We found haemorrhage, formation of interstitial oedema in alveolar septa, and infiltration of the perivascular space with erythrocytes and granulocytes (Figure 2A,B). Higher doses of bacteria led to earlier onset of the described pathophysiological changes, especially swelling and thickening of the alveolar septa. When using 10^8^ cfu ExoY bacteria per mouse, tissue alterations were detectable after 4 h. With 10^7^ cfu per mouse, first signs of histological changes could be observed with a delay of 4 h. However, infection with ExoY^K81M^ only caused mild inflammation and general signs of bacterial infection, such as a temporary infiltration with neutrophils (for high-resolution representative images of mice lungs infected with 10^7^ or 10^8^ cfu of ExoY^K81M^ or ExoY at 2, 8, and 48 h post infection, see Supplementary Figures S1–S3). After 24 to 48 h, mice lungs infected with 10^7^ cfu ExoY^K81M^ were almost free from neutrophils and the above-mentioned signs of infection. Mock-infected mice (PBS) showed neither granulocyte infiltration nor other inflammatory signs, but only a slight oedematous swelling of the alveolar septa (Supplementary Figure S4). On the contrary, residual neutrophils and slight oedema were observed in lungs infected with 10^8^ cfu of ExoY^K81M^ (Figure 2A,B). Significant differences between the ExoY and ExoY^K81M^ infection were also found in the number of infiltrating neutrophils in the lung tissue. At 2–8 h post infection both ExoY- and ExoY^K81M^-infected lungs showed infiltration with neutrophils (see Supplementary Figures S1 and S2). However, while ExoY^K81M^-infected lung tissue had largely been cleared of neutrophils after 24–48 h post infection, neutrophils persisted in ExoY-infected lungs (Figure 2A,B and Figure S3).

We analysed local cytokine concentrations in BAL-f (Figure 3) and systemic cytokine concentrations in sera of ExoY- or ExoY^K81M^-infected mice (Figure 4). Both the infection with ExoY and ExoY^K81M^ led to a local and systemic unspecific inflammatory cytokine response. Cytokine secretion and enrichment were dependent on the bacterial infection dose, with overall lower cytokine concentrations being detected in mice infected with 10^8^ cfu per mouse. In these mice neither local, nor systemic, cytokine concentrations differed significantly between ExoY-infected and ExoY^K81M^-infected mice. At lower infection doses, significant differences in height and kinetics of cytokine concentrations in BAL-f were mostly confined to the time point around the maximum cytokine response (2–4 h for IL-1β and Ccl3, 2–8 h for TNF, 8–24 h for Cxcl-1, Ccl2). Whereas Cxcl1/Ccl2 concentrations were increased in BAL-f of ExoY-infected mice, IL-1β, TNF, and Ccl3 were decreased as compared to ExoY^K81M^-infected mice (Figure 3). 

In sera, only the peak concentrations of Cxcl1 (8 h), Ccl2 (8 h), Ccl3 (8 h), IL-1β (4 h), and IL-6 (4–8 h after infection) were significantly elevated in ExoY-infected mice as compared to ExoY^K81M^-infected mice (Figure 4). Supplementary Figures S5 and S6 show the same data with discontinuous ordinate axes for a better evaluation of the cytokine response in mice infected with 10^8^ cfu.

### 2.2. Apoptosis and Disruption of the Epithelial Barrier

**Figure 5:**
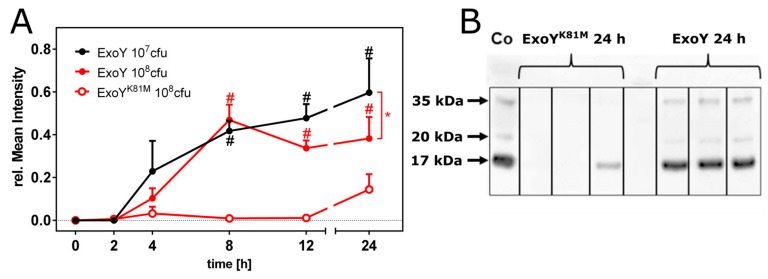
Break of the epithelial barrier in infected lungs. (**A**) Time-dependent concentrations of the serum protein transthyretin in BAL fluid of ExoY-infected (red/black filled circles), and ExoY^K81M^-infected (open red circles) mice. Protein abundance was estimated by normalizing the mean grey value of the respective Western blot band to the mean grey value of the control band (mouse serum). Data represent the mean ± SD of *n* = 6 animals. Differences between ExoY infection doses (*), or between 10^8^ cfu ExoY and 10^8^ cfu ExoY^K81M^ (#) were considered significant when *p* ≤ 0.05 (two-way-ANOVA with post-hoc Holm-Sidak correction). The ExoY^K81M^ group is missing due to limited sample material; and (**B**) representative Western blot of transthyretin in BAL-f of mice infected with 10^8^ cfu of ExoY or ExoY^K81M^. Co = serum control. Transthyretin is detectable at ~15 kDa in the serum control and in significant amounts in BAL-f of ExoY-infected mice.

To identify the precise beginning of tissue destruction, haemorrhage and disruption of the pulmonary barrier, we analysed the concentration of the exclusive serum protein transthyretin, a precursor form of albumin, in the BAL-f of mice infected with ExoY or ExoY^K81M^ (Figure 5A). Interestingly, transthyretin was detectable in the BAL-f of ExoY-infected mice as early as after 4 h post infection, but was not detectable in significant amounts in ExoY^K81M^-infected mice until 24 h after infection (Figure 5A,B).

**Figure 6:**
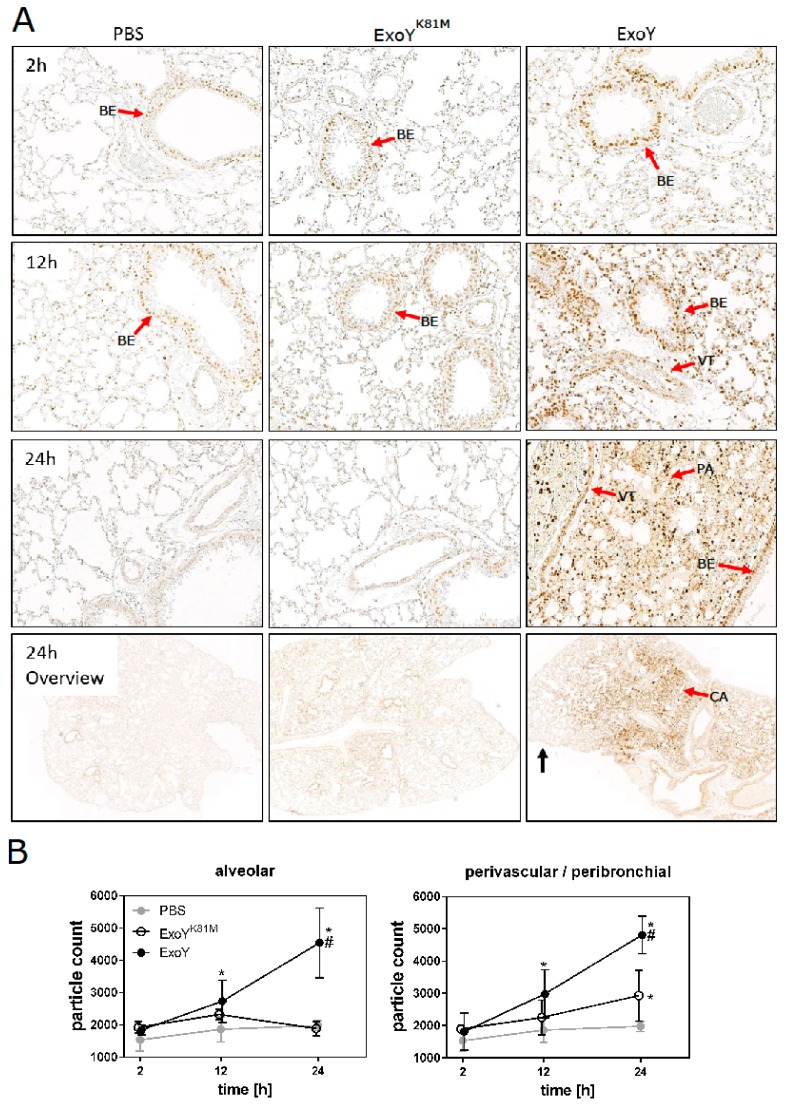
Occurrence of cell death in infected lungs. (**A**) Immunohistochemistry (anti-ssDNA) of lung sections counterstained with haematoxylin 24 h after infection with *P. aeruginosa* strain ExoY^K81M^ (10^7^ cfu/mouse) or ExoY (10^7^ cfu/mouse). The red arrows point at the bronchial epithelium (BE), vascular tissue (VT), parenchyma (PA), and central airways (CA). The black arrow points at the distal airways of the lung. Scale bar = 200 µm. (**B**) Quantification of cells stained positive with anti-ssDNA in alveolar and perivascular tissue (ImageJ). Differences between experimental groups and PBS (*), and between ExoY and ExoY^K81M^ (#) were considered significant when *p* ≤ 0.05 (two-way-ANOVA with post-hoc Holm-Sidak correction).

In ExoY-infected lungs, apoptotic cells (red arrows, Figure 6A) were first detected in bronchial epithelium, but over time apoptotic cells were detected even in the alveolar space (black arrow, Figure 6A). Finally, apoptotic cells were observed in pulmonary parenchyma and vascular tissue and, thereby, literally all cell types of the lung were affected by ExoY after 24 h. In contrast, infection with the ExoY^K81M^ mutant only affected the bronchial epithelium: 24 h after infection, endothelial cells and lung parenchyma were still largely unaffected by apoptosis and bronchial epithelium already partially recovered (Figure 6A, Overview). These differences in the distribution of apoptotic cells between ExoY- and ExoY^K81M^-infected lungs are confirmed by automatic quantification of apoptotic cells (Figure 6B).

## 3. Discussion

**Figure 7:**
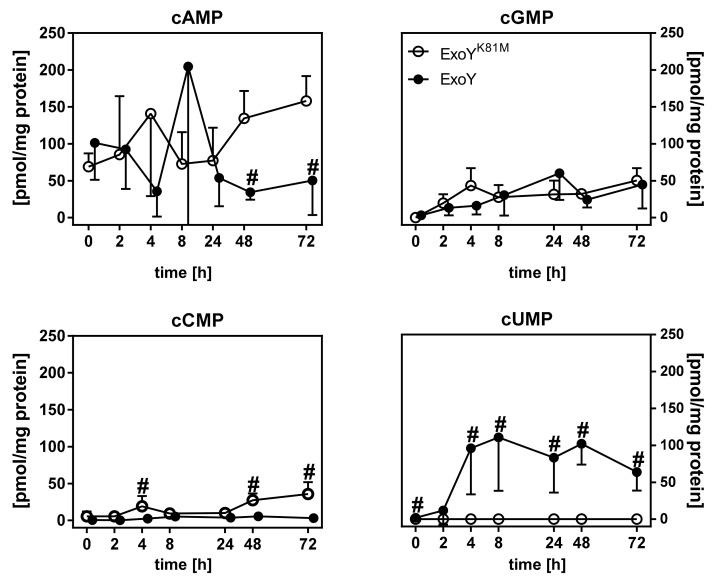
Occurrence of cyclic nucleotides in mouse lungs infected with 10^6^ cfu of ExoY or ExoY^K81M^ (adapted from previously-published work [14]; please note that the data were generated using the very same experimental animals). Concentrations of cyclic nucleotides in lung homogenates were measured mass-spectrometrically and normalized to total protein content. The LLOQ for standard cAMP was 0.04 pmol/sample, for standard cGMP 0.07 pmol per sample, for standard cCMP 0.07 pmol/sample, and for standard cUMP 0.4 pmol per sample [20]. Differences between ExoY and ExoY^K81M^ were considered significant (#), when *p* ≤ 0.05 (multiple t-tests with post-hoc Holm-Sidak correction).

The most important finding of our study is that the first significant occurrence of cUMP in infected mouse lungs after 2–4 h, as already published before [14,20] (Figure 7), coincides both with a significantly-altered early innate immune response (predominantly IL-1β, TNF, and Ccl3; see Figure 3 and Figure 4) and the onset of lung barrier integrity loss as detected by transthyretin leakage into BAL-f (see Figure 5). The pathogenic effect of ExoY has already been correlated with the prolonged increase of cAMP and cGMP concentrations affecting microtubular dynamics in endothelial cells [21,22,23], but little is known about the acute effects of ExoY and the generated cyclic nucleotides, especially cUMP, during very early infection. 

In a rat model of acute pneumonia, both ExoY and ExoY^K81M^ led to a lung infection with neutrophilic infiltrates and tissue consolidation, but only ExoY caused severe alveolar oedema/haemorrhage and a delayed lung repair [17]. Not only could we reproduce the findings of the rat model in which infection with ExoY, but not ExoY^K81M^, led to a progressive haemorrhagic alveolar lung oedema, but we also provide evidence that these differences of the infectious phenotype already occur at very early time points prior to 48 h after infection (see Table 1). 

Within these 48 h, both ExoY- and ExoY^K81M^-infected mice showed distinct signs of infection in our study, but, histologically, only ExoY led to severe lung tissue alterations, whereas ExoY^K81M^ caused only mild signs of inflammation, such as a moderate and temporary infiltration of the lung with neutrophilic granulocytes and a mild interstitial oedema. Again, this corresponds well with the published data from a rat model [17] and points toward an early (2–48 h) role of catalytically-active ExoY as an oedema factor and inductor of increased lung barrier permeability for fluids, and even cells.

Both body weight and body temperature of the mice decreased during the infection reflecting the reduced motility due to the severe illness of the mice. The massive body weight loss, especially in the early hours, was most probably due to a reduced water intake of the ill animals, as the urine of the animals became more and more scarce and concentrated over time (observation of the experimenters). Of all parameters, the lung score we applied was most appropriate for differentiating between ExoY- and ExoY^K81M^-infected mice, indicating that the most profound immunological effects of ExoY can be found in the lung.

Indeed, we found the proinflammatory cytokines IL-1β, TNF, and Ccl3 reduced in BAL-f of ExoY-infected mice as compared to ExoY^K81M^-infected mice at 2–4 h after infection. During this early innate immune response, the stage is set for an effective mount of a host immune response against *P. aeruginosa* [24,25]. It has been documented that ExoY lowers concentrations of TNF and IL-1β after infection of rat lungs or epithelial cells, respectively [26,27]. Again, our data indicate that this dampening of the proinflammatory immune response within the infected organ already starts at 2-4 h after infection. All measured proinflammatory cytokines in the sera of infected mice, however, were increased in ExoY-infected mice in our study, which corresponds well with the overall high disease severity. A massive hypercytokinemia as a sign for a severe inflammatory response syndrome already occurred at the lower infection dose of 10^7^ cfu/mouse. The overall reduction of cytokine concentrations at 10^8^ cfu/mouse may, thus, already be due to immune paralysis caused by the overwhelming bacterial load. The potent neutrophil attractant Cxcl-1 was elevated in BAL-f of ExoY-infected mice. This may be an explanation for the progressive destruction and infiltration of ExoY-infected lungs with neutrophilic granulocytes and, consequently, for the progression of lung oedema and haemorrhage, since the radical oxygen species (ROS) and serine proteases produced by activated neutrophils can contribute to lung injury during acute infections [28,29]. Not only may the increased production of ROS and serine proteases be accountable for tissue damage, but the bacterium, or even ExoY itself, may induce cell death within host tissues. Cells infected with *P. aeruginosa* expressing a functional T3SS have been shown to upregulate CD95/CD95L, which—upon activation—induces apoptosis [30]. In addition, it has been shown that high intracellular concentrations of cUMP can stimulate apoptosis [31,32,33]. An excessive incidence of cell death in the lung leads to a breakdown of lung barrier integrity. Indeed, we detected the serum protein transthyretin in BAL-f of ExoY-infected mice already at 4 h after infection, indicating a compromised blood-air barrier. This, again, coincides with the onset of cUMP accumulation. 

The higher prevalence and wide-spread tissue distribution of apoptotic cells in the ExoY-infected lungs may be due to the steadily high cUMP concentrations, the increased ROS/protease concentrations and the loss of barrier integrity with facilitated invasion of bacteria. Most likely, all these effects contribute to the aggravation of lung tissue destruction by the effector protein culminating in the detection of apoptotic cells even in alveolar tissue of ExoY-infected mice, whereas ExoY^K81M^ mice have largely recovered 24 h after infection.

One caveat of our current study is that the genetically-engineered *P. aeruginosa* strains we used are both overexpressing the effector proteins ~20-fold. Thus, natural strains expressing ExoY may not show the pronounced clinical symptoms we observed in our study [14]. However, since there is currently no appropriate model system to reveal specific effects of ExoY in natural doses, the use of the engineered strains is a necessary prerequisite to further elucidate the function of ExoY.

The cytokine and cNMP concentrations detected in our study show noticeable variation. This may be caused, on the one hand, by inherent fluctuations typical for complex biological organisms. On the other hand, the complexity of the infection model may add variation to the data. Due to the limited size (*n* = 6) of our experimental groups our data may be affected by these sources of variation and we may have missed more subtle effects as a consequence of statistical underpowerment.

A limitation of our study is that, although we have used defined infection doses, we cannot rule out the possibility that the observed effects are confounded by differential growth and distribution characteristics of the used strains in the host organism. Recently, He et al. have provided evidence for an increased bacterial load in mouse lungs infected with *P. aeruginosa* strain PAO1 at two days or four days after infection as compared to an ExoY-deficient PAO1Δ*exoY* strain [27]. Using this system, however, one cannot conclude whether or not the NC activity of ExoY affects bacterial growth. Furthermore, due to the expression of the T3SS effector proteins ExoS and ExoT in PAO1 [34], the data are not comparable to ours (our PA103 strain only expresses ExoY, but neither ExoS, nor ExoT, nor ExoU), because the influence of ExoY on the bacterial load may be modulated by the presence of other T3SS effector proteins. However, regardless if there are differences in bacterial growth of ExoY and ExoYK81M in infected lungs, they unequivocally depend on the NC activity of ExoY in our model. Thus, the effects observed in this study are due to the NC activity of ExoY, no matter whether they are direct or indirect.

## 4. Conclusions

In summary, our data indicate a role of the catalytically-active ExoY in effectively targeting the distal airways by impairing early innate immune response and triggering apoptosis. This may enable the bacterium to penetrate into the lung parenchyma and systemic circulation. To prove this, a thorough investigation of the bacterial distributions in infected lungs and of the kinetics of the bacterial burden in the tissue is urgently needed. Furthermore, the mechanistic link between the consistently high cUMP concentrations in infected mice lungs and the associated pathophysiologic changes (Table 1) still remains elusive. Thus, in future studies we will investigate the receptors and signal transduction processes mediating the observed cUMP effect.

## 5. Materials and Methods

### 5.1. Chemicals and Reagents

If not stated otherwise, all chemicals and reagents were obtained from Sigma–Aldrich, Munich, Germany. Phosphate-buffered saline (PBS), DMEM/F12 solution (Dulbecco’s modified Eagle’s medium/nutrient mixture F-12 Ham), DMEM, and RPMI-1640 were purchased from Gibco^®^ (Life Technologies, Darmstadt, Germany). Ketamine was obtained from Dr. E. Gräub, Bern, Switzerland, and midazolam was obtained from Bayer, Wuppertal, Germany. Haematoxylin and eosin were purchased from Merck, Darmstadt, Germany. Blocking reagent Roti-Block was purchased from Carl Roth, Karlsruhe, Germany.

### 5.2. Bacterial Strains and Cultivation of Bacteria

We used the *P. aeruginosa* strains PA103∆exoUexoT::Tc pUCPexoY (“ExoY”) and PA103∆exoUexoT::Tc pUCPexoY-K81M (“ExoY^K81M^”). Bacteria were stored as glycerol stocks at −80 °C. Bacteria were streaked on Vogel Bonner (VB) medium agar plates containing 400 µg/mL carbenicillin and incubated at 37 °C for 16 h. The next day, a large loopful of bacteria was suspended in PBS and the number of colony forming units (cfu)/mL was determined by measuring the optical density at a wavelength of 600 nm (OD600) with the Ultrospec 10 cell density meter (GE Healthcare, Buckinghamshire, UK), OD600 = 0.25 ≈ 2 × 10^8^ cfu/mL. Desired bacterial concentrations were adjusted by serial dilution with PBS and checked on VB dose control plates. A brief description of the bacterial strains used in this study is shown in Table 2.

### 5.3. Animal Experiments and Infection

Animal housing and experimental procedures were approved by the animal welfare committee of the Hannover Medical School, complied with the German animal welfare legislation and were finally approved by the Lower Saxony State Office for Consumer Protection and Food Safety (LAVES, date of approval: 19 August 2013, AZ 33.14-42502-04-13/1196). Female C57BL/6J mice (aged 8–10 weeks; body weight, ~20 g) were purchased from Janvier Labs and housed in the animal facility of Hannover Medical School. Mice were intratracheally instilled with 10^7^–10^8^ cfu of the nucleotidyl cyclase-active ExoY *P. aeruginosa* strain ExoY or the inactive ExoY strain ExoY^K81M^ [10] according to the previously-described protocols [14,19]. Mock-infected mice were instilled with the equivalent volume (50 µL) of PBS only. For intraperitoneal (i.p.) anaesthesia, 5 mL midazolam (1 mg/mL) was mixed with 1 mL ketamine (100 mg/mL) and filled up to 10 mL with 0.9% (*w*/*v*) aqueous NaCl solution. A dose equivalent to 0.1 mL/10 g body weight was injected intraperitoneally. To prevent salivation, 0.1 mg atropine sulphate per kg body weight was injected subcutaneously. Mice were sacrificed by an overdose of aesthetic (1 g/kg ketamine, 100 mg/kg xylazine), 0–48 h post-infection. Blood was taken by puncturing the right heart ventricle and bronchoalveolar lavage (BAL) was performed via the trachea using 1 mL PBS. Individual lung lobes were weighed and used for different analyses (mass spectrometry analysis of cyclic nucleotides, histology/immunohistochemistry for single-stranded DNA, cytokine protein analyses).

### 5.4. Disease Score, Body Temperature, Body Weight, and Lung Score

After initial infection, mice were monitored at defined time points (0, 2, 4, 8, 12, 24, and 48 h) regarding their rectal temperature and their body weight. The overall health was assessed by a multiparametric disease score as described before [19]. In brief, vocalization, piloerection, posture, locomotion, breathing, curiosity, nasal secretion, grooming, and dehydration were recorded and dysfunctions were scored with zero, one or two points. Summing up these points resulted in the following score: unaffected (0–1); slightly affected (2–4); moderately affected (5–7); severely affected (8–10); or moribund (≥11). Inflammation of infected lungs was assessed using a macroscopic lung pathology score ranging from zero to two points (no pathologic alteration = 0, mild pathologic changes = 1, severe pathologic changes = 2). Scoring was performed for blood in BAL-f (0–2), visual lung tissue anomalies (0–2), thoracic bleeding (1) and signs of systemic affection (2), yielding a sum score ranging from 0–7.

### 5.5. Histology

Lungs of mice were harvested 0, 2, 4, 8, 12, 24, and 48 h after infection, fixed with 4% (*v*/*v*) formalin, and embedded in paraffin. The paraffin blocks were cut into 4 mm thick slices and stained with haematoxylin/eosin. Microphotographs were taken using a Zeiss AxioVert 200M microscope (Carl Zeiss Microscopy GmbH, Jena, Germany). 

### 5.6. Measurement of Cytokine Concentrations

Cytokines in BAL-f and sera were measured by Multiplex Luminex^®^ Assays (R&D Systems, Abingdon, UK). A customized, 6-plex, validated for the used body fluids, was used, the cytokine panel included Tnf, Ccl3, Ccl2, Il-1β, Il-6, and Cxcl1. Assay data were analysed as recommended by the manufacturer. 

### 5.7. Western Blot Detection of Transthyretin (TTR) Levels in BAL-f

TTR concentrations in BAL-f of infected mice and in serum of a healthy control subject (positive control) were evaluated by Western blot analysis. Samples of 17 µL BAL-f and 4 μL serum were separated electrophoretically on 15% sodium dodecyl sulphate-polyacrylamide gels. The proteins were electrically transferred to PVDF membranes (Roche, Mannheim, Germany). Membranes were blocked for 1 h at RT in Roti-Block and incubated with primary antibodies against TTR (mouse monoclonal antibody #PA5-27220, 0.33 µg/mL; Fisher Scientific, Waltham, MA, USA) overnight at 4 °C. Then, membranes were incubated with a rat monoclonal anti-mouse IgM antibody (HRP-conjugated, 2 µg/mL; Zymed, Vienna, Austria) at RT for 1 h. Protein levels of the BAL-f were analysed by evaluation of the mean intensities of the western blot bands in relation to the protein level of the serum defined as 100% (ImageStudio v5.0, Li-Cor, Bad Homburg, Germany).

### 5.8. Immunoassay for Single-Stranded DNA in Apoptotic Cells

The apoptosis assay is based on the selective denaturation of DNA in condensed chromatin of apoptotic cells and the detection of denatured DNA with a monoclonal antibody highly specific for single-stranded DNA. The assay detects apoptotic, but not necrotic, cells or cells with DNA breaks in the absence of apoptosis. Formalin-fixed paraffin-embedded tissues were dewaxed using xylol and alcohol followed by a digestion with a 5% (*w*/*v*) saponine solution and proteinase K. Afterwards, lung tissues were incubated with warm formamide and quenched by 3% (*v*/*v*) H_2_O_2_ solution. Optimal results were obtained at a relatively low temperature (56 °C) in the presence of formamide. Slices were blocked with 3% (*w*/*v*) milk powder, then incubated with the primary antibody (mouse mAb against ssDNA, #ALX-804-192-L001, Enzo Life Science, Lörrach, Germany) overnight, washed and stained with the secondary antibody (rat monoclonal anti-mouse IgM antibody, HRP-conjugated, 2 µg/mL; Zymed, Vienna, Austria) for 1 h and then developed for 10 min with 3,3′-diaminobenzidine (DAB)-plus substrate (Zymed, Vienna, Austria). Lung tissue was counterstained with haematoxylin solution according to Mayer for 15 s. For quantitative analysis, micrographs were evaluated by applying automatic particle counting using Fiji application v1.51h [35]. Particles were defined as positive when they exceeded a threshold of 140 in black/white transformed images. Six images per slice (magnification 20×, one slice/mouse) were analysed, three images showing only the alveolar space and three showing the perivascular space. In each group there were six mice per time point.

### 5.9. Statistics

Data are presented as means ± SD of *n* = 6 animals (animal studies) or based on 3–4 independent experiments performed in technical duplicates. GraphPad Prism 6.0 (San Diego, CA, USA) was used for the calculation of means and SD. Statistical significances were inferred using either multiple *t*-tests or two-way-ANOVA, each with post-hoc Holm-Sidak correction for multiple comparisons. An adjusted *p*-value of <0.05 was considered as statistically significant.

## Figures and Tables

**Table 1:** Characteristic differences between ExoY and ExoY^K81M^ in the acute mouse lung infection model. A moderate or strong increase of the parameter value is marked as “↑” or “↑↑”, respectively. An assessment of the measured parameters’ kinetics is shown in the second subcolumn of each experimental group. TTR = transthyretin, BAL-f = bronchoalveolar lavage fluid, n.d. = not detectable.

Parameter	ExoY		ExoY^K81M^	
cUMP	↑↑	4–72 h	↔	n.d.
cCMP	↔	constantly low	↑	48–72 h
cGMP	↑	no difference to ExoY^K81M^	↑	no difference to ExoY
cAMP	↑	transient rise at 8 h	↑↑	48-72 h
apoptosis/pyroptosis	↑↑	progressive	↑	recovery
TTR in BAL-f	↑	Onset at 4 h	↔	slight increase after 24 h
disease severity	↑↑	mice had to be sacrificed at t > 24 h	↑	recovery
interstitial edema	↑↑	progressive	↑	recovery
alveolar edema/hemorrhage	↑↑	progressive	↔	none
neutrophilic infiltration	↑↑	progressive	↑	recovery
serum proinflammatory cytokines	↑↑	4 h (IL-1β); 8 h (Cxcl1, Ccl2, Ccl3, IL-6, TNF)	↑	8 h (Cxcl1, Ccl2, Ccl3, IL-1β IL-6); 24 h (Ccl3, TNF)
IL-1β, Ccl3, TNF in BAL-f	↑	2–4 h	↑↑	2–4 h
Cxcl1, Ccl2, IL-6 in BAL-f	↑↑	8–12 h	↑	4–8 h (Cxcl1, IL-6), 24 h (Ccl2)

**Table 2:** Bacterial strains used in this study.

Strain	Strain Name	Source	Virulence	ExoS/T/U	ExoY
PA103∆exoUexoT::Tc pUCPexoY	ExoY	Genetically engineered [10]	+	−	+
PA103∆exoUexoT::Tc pUCPexoY-K81M	ExoY^K81M^	Genetically engineered [10]	−	−	+ (loss-of-function mutation K81M)

## Supplementary Materials

The following are available online at http://www.mdpi.com/2072-6651/10/5/185/s1; Figure S1: Representative micrographs of ExoY-/ExoY^K81M^-infected mouse lungs 2 h after infection, Figure S2: Representative micrographs of ExoY-/ExoY^K81M^-infected mouse lungs 8 h after infection, Figure S3: Representative micrographs of ExoY-/ExoY^K81M^-infected mouse lungs 48 h after infection, Figure S4: Representative micrograph of mouse lungs (PBS control) 24 h after infection. Figure S5: Expression of inflammatory cytokines in BAL-f of mice after infection with ExoY^K81M^ and ExoY, Figure S6: Expression of inflammatory cytokines in sera of mice after infection with ExoY^K81M^ and ExoY.
