# Supplementary material for: The Role of Pseudomonas aeruginosa ExoY in an Acute Mouse Lung Infection Model
Source: Toxins (Basel). 2018 May 4;10(5):185. doi: 10.3390/toxins10050185 (PMC5983241; doi:10.3390/toxins10050185)
Supplement: Supplementary file 1 [file toxins-10-00185-s001.pdf]

Supplementary Materials: The role of Pseudomonas aeruginosa ExoY in an Acute Mouse Lung Infection Model

Christina Kloth, Bastian Schirmer, Antje Munder, Tane Stelzer, Justin Rothschuh and Roland Seifert

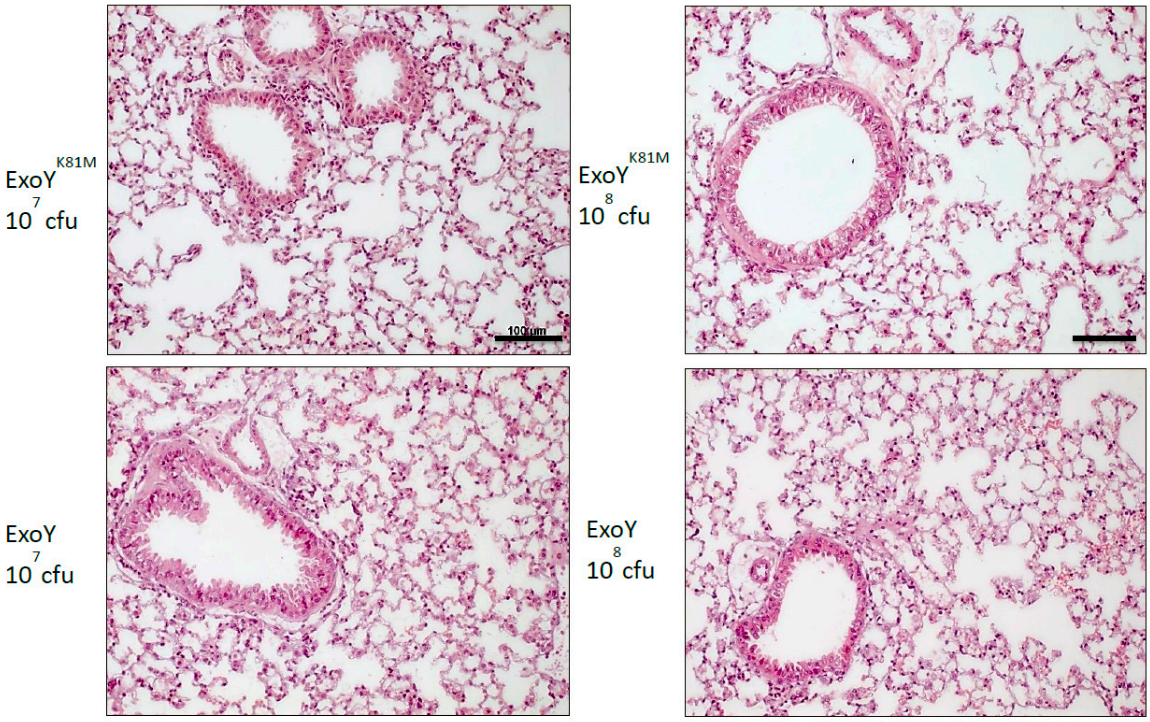

Figure S1. Representative micrographs of ExoY/ExoY^{K81M}-infected mouse lungs 2 h after infection. Standard H&E staining, scale bar = 100 µm.

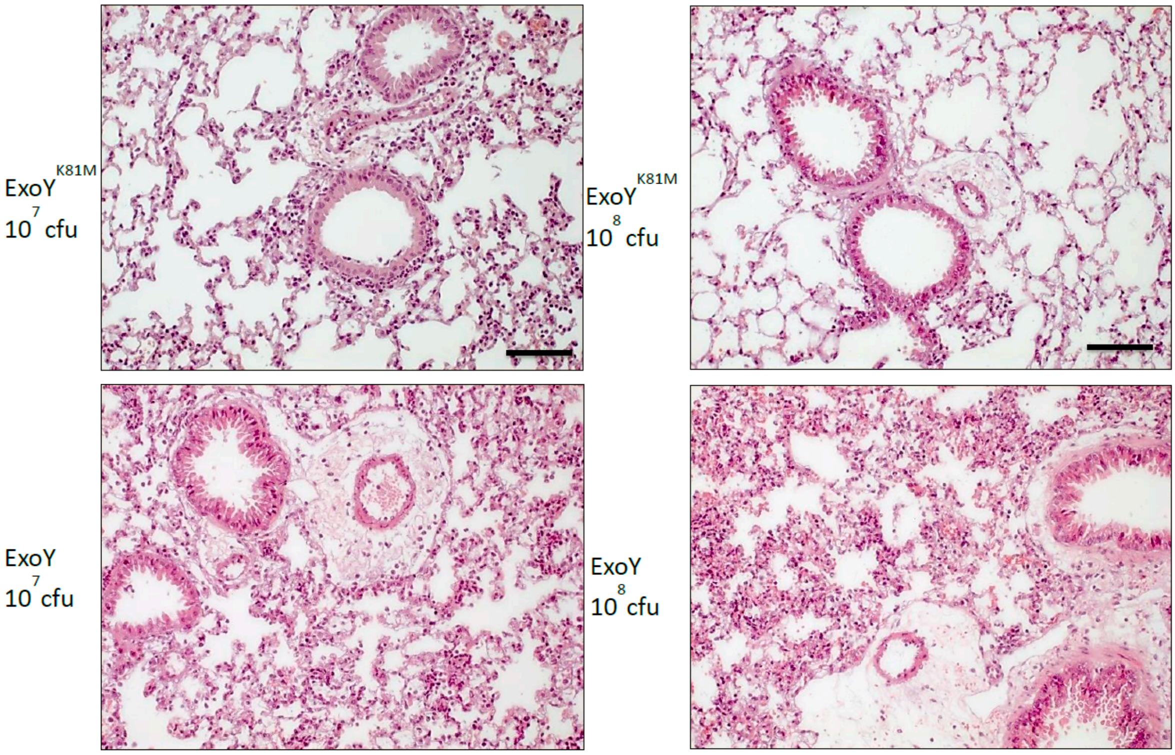

Figure S2. Representative micrographs of ExoY/ExoY^{K81M}-infected mouse lungs 8 h after infection. Standard H&E staining, scale bar = 100 µm.

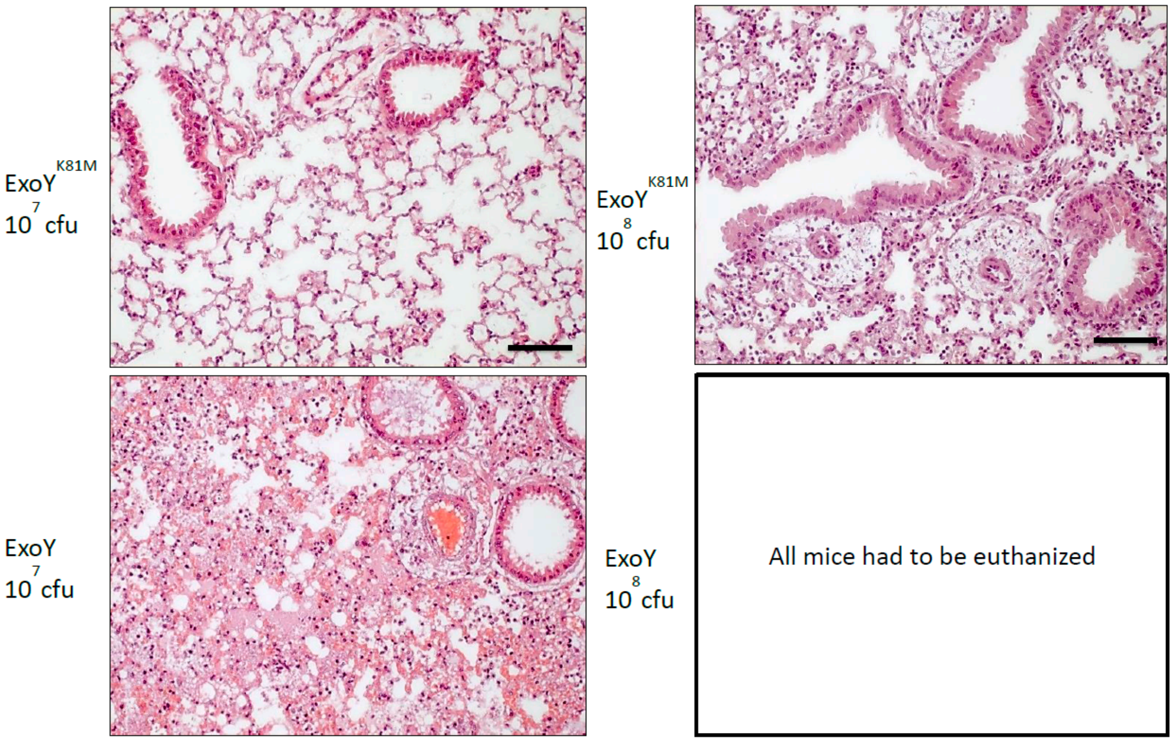

Figure S3. Representative micrographs of ExoY/ExoY^{K81M}-infected mouse lungs 48 h after infection. Standard H&E staining, scale bar = 100 µm. All mice infected with 108 cfu of ExoY had to be sacrificed because they met the pre-defined termination criteria.

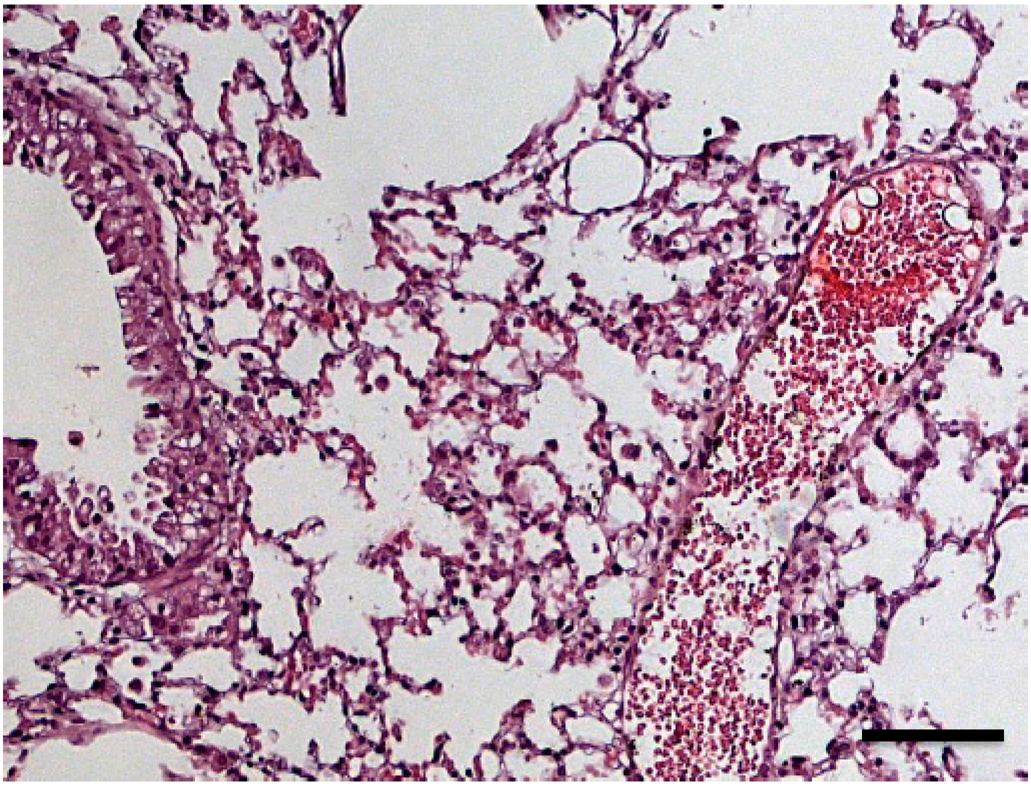

Figure S4. Representative micrograph of mouse lungs (PBS control) 24 h after infection. Standard H&E staining, scale bar = 100 μm.

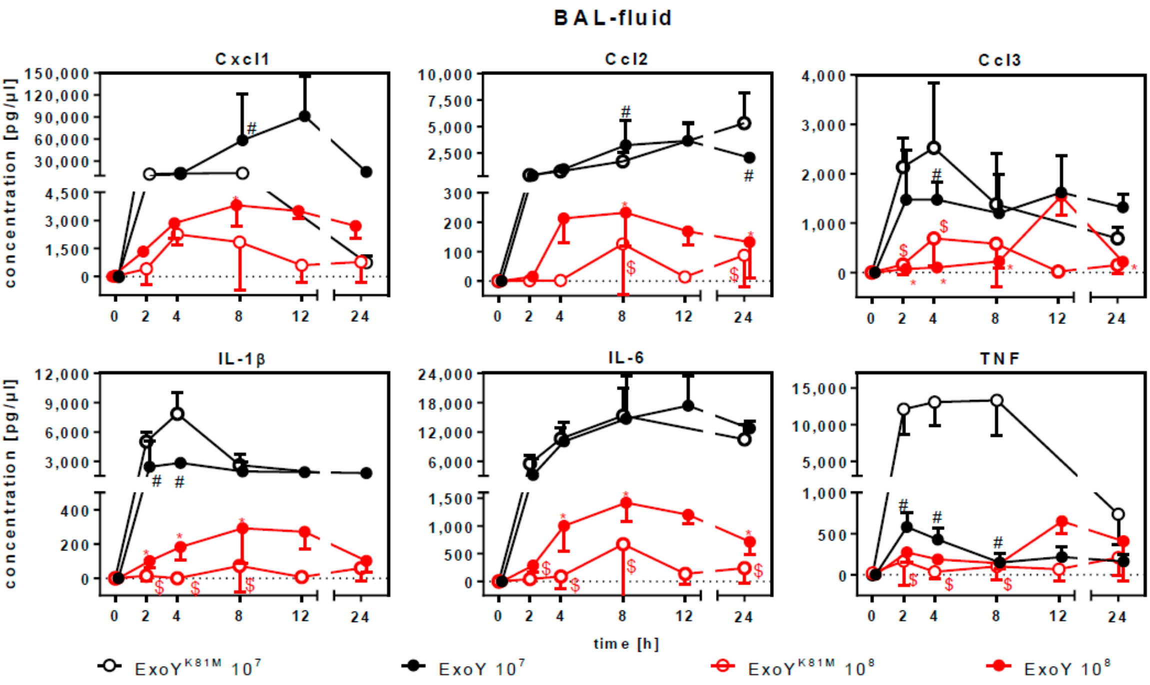

Figure S5. Expression of inflammatory cytokines in BAL-f of mice after infection with ExoY^{K81M} and ExoY. The same data as presented in Fig. 3 are presented here with segmented ordinates in order to allow a better evaluation of the experimental groups infected with 108 cfu. Data represent the mean ± SD of *n* = 6 animals. Differences between ExoY infection doses (*), between ExoYK81M infection doses ($), and between ExoY and ExoYK81M of same infection dose (#) were considered significant, when p ≤ 0.05 (two-way-ANOVA with post-hoc Holm-Sidak correction).

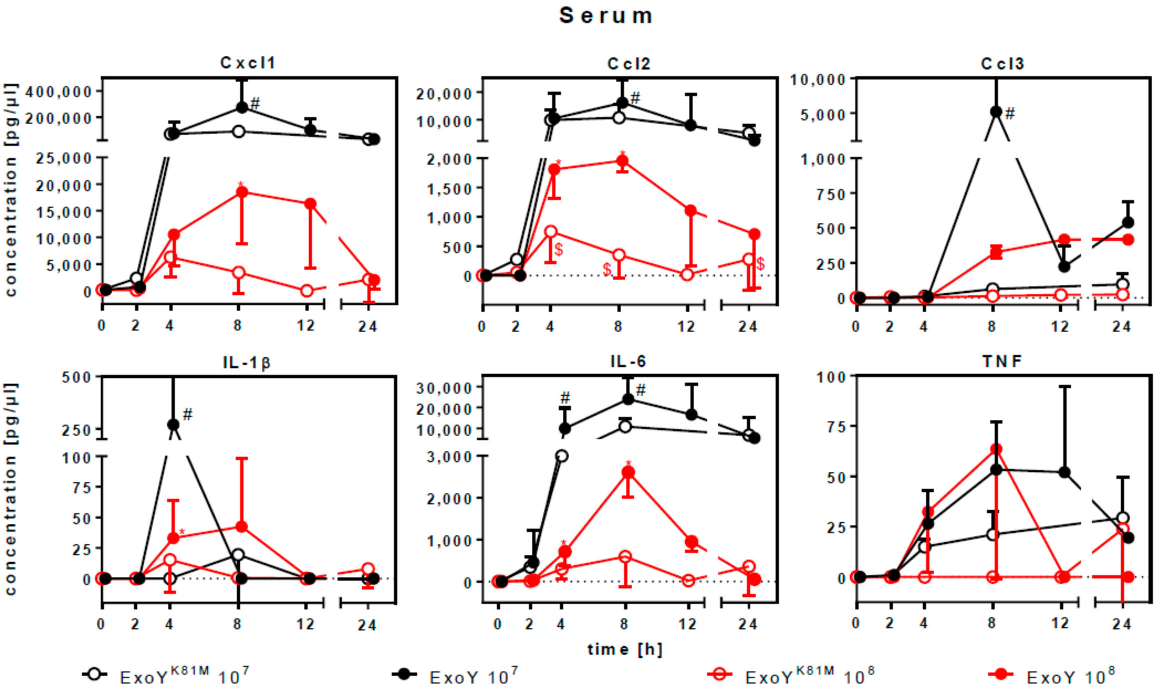

Figure S6. Expression of inflammatory cytokines in BAL-f of mice after infection with ExoY^{K81M} and ExoY. The same data as presented in Fig. 4 are presented here with segmented ordinates in order to allow a better evaluation of the experimental groups infected with 108 cfu. Data represent the mean ± SD of n = 6 animals. Differences between ExoY infection doses (*), between ExoYK81M infection doses ($), and between ExoY and ExoYK81M of same infection dose (#) were considered significant, when $p \leq 0.05$ (two-way-ANOVA with post-hoc Holm-Sidak correction).
